# Deciphering the protective effect of Buzhong Yiqi Decoction on osteoporotic fracture through network pharmacology and experimental validation

**DOI:** 10.1186/s13018-023-03545-7

**Published:** 2023-02-04

**Authors:** Zhen Hua, Shijie Dai, Shaoshuo Li, Jianwei Wang, Hongcheng Peng, Yi Rong, Hao Yu, Mingming Liu

**Affiliations:** 1 Department of Orthopedics, Wuxi Hospital of Traditional Chinese Medicine, Wuxi, China; 2 College of Pharmacy, Zhejiang Chinese Medical University, Hangzhou, Zhejiang China; 3 Department of Orthopedics, The Second People’s Hospital of Lianyungang, 41 Hailian East Road, Haizhou District, Lianyungang, 222006 Jiangsu Province China

**Keywords:** Buzhong Yiqi Decoction, Osteoporotic fracture, Network pharmacology

## Abstract

**Background:**

Osteoporotic fracture (OPF) is one of the most common skeletal diseases in an aging society. The Chinese medicine formula Buzhong Yiqi Decoction (BZYQD) is commonly used for treating OPF. However, the essential bioactive compounds and the underlying molecular mechanisms that promote fracture repair remain unclear.

**Methods:**

We used network pharmacology and experimental animal validation to address this issue. First, 147 bioactive BZYQD compounds and 32 target genes for treating OPF were screened and assessed. A BZYQD-bioactive compound-target gene-disease network was constructed using the Cytoscape software. Functional enrichment showed that the candidate target genes were enriched in oxidative stress- and inflammation-related biological processes and multiple pathways, including nuclear factor kappa B (NF-κB), and mitogen-activated protein kinase (MAPK) signaling pathways. Furthermore, an OPF rat model was established and treated with BZYQD.

**Results:**

The results revealed that BZYQD ameliorated OPF characteristics, including femoral microarchitecture, biomechanical properties, and histopathological changes, in a dose-dependent manner. Results of enzyme-linked immunosorbent assay showed that BZYQD reduced the serum’s pro-inflammatory cytokines [Tumor necrosis factor-alpha (TNF-α), Interleukin (IL)-1β, and IL-6] and improved oxidative stress-related factors [glutathione (GSH) and superoxide dismutase (SOD)]. BZYQD significantly decreased the protein expression of NF-κB in OPF rat femurs, suppressed NF-κB activation, and activated the nuclear factor-erythroid factor 2-related factor (Nrf2)/heme oxygenase 1 (HO-1) and p38 MAPK as well ERK pathways.

**Conclusions:**

Our results suggest that BZYQD could improve inflammation and oxidative stress during fracture repair by suppressing NF-κB and activating Nrf2/MAPK signaling pathways.

## Introduction

Osteoporosis (OP) is a systemic skeletal disease caused by low bone mass and the destruction of bone microstructure [1]. It is more common in the elderly, with a higher incidence in postmenopausal women [2]. Osteoporotic fracture (OPF) is a severe traumatic fracture caused by OP and is characterized by decreased bone mass and deterioration of the bone trabecular structure [3]. OPF frequently occurs in the vertebral body, proximal femur, distal humerus, and distal radius and is associated with high morbidity and disability [4]. About 2.33 million patients with OPF were reported in China in 2010, and the number is expected to reach 5.99 million by 2050 [3]. Therefore, OPF is not only a public health problem but also a social problem.

Successful fracture healing depends on inflammation, angiogenesis, and chondrogenesis, including osteoblast recruitment, migration, and homing [5]. An imbalance in bone remodeling, characterized by bone formation by osteoblasts and bone resorption by osteoclasts, is the cause of many skeletal diseases, including OP [6]. The fracture healing process is complex and requires multi-stage integration of genetic, cellular, and physiological factors [7]. Under the regulation of these molecules, bone is remodeled through intramembranous or endochondral ossification, during which osteoblast proliferation and apoptosis control are critical factors in enhancing bone repair [8, 9]. Therefore, identifying strategies to modulate osteoblasts and osteoclasts during fracture healing may be to facilitate fracture healing.

The efficacy of traditional Chinese medicine (TCM) has been confirmed in the long history of clinical diagnosis and treatment at different stages of fracture repair [10, 11]. Buzhong Yiqi Decoction (BZYQD) is a classical Chinese herbal formula initially recorded in “Piwei Lun” (medical literature written in Jin Dynasty, AD 1247). BZYQD has the effects of raising the kidney’s Yang, removing blood stasis, invigorating the spleen and stomach, and strengthening bones; it has been commonly used for treating fractures [12]. BZYQD is composed of *Atractylodes macrocephala Koidz* (Largehead Atractylodes Rhizome, Baizhu), *Radix Bupleuri* (Bupleurum chinense, Chaihu), *Citrus reticulata* (Dried tangerine, Chenpi), *Ziziphus jujuba* Mill (Jujubae Fructus, Dazao), *Angelicae Sinensis Radix* (Chinese Angelica, Danggui), *Codonopsis pilosula (Franch.) Nannf.* (Codonopsis Radix, Dangshen), *Astragalus membranaceus* (Fisch.) Bunge (Hedysarum multijugum Maxim, Huangqi), *Cimicifugae rhizoma* (Largetrifoliolious bugbane rhizome, Shengma), *Zingiber officinale Roscoe* (Ginger, Shengjiang), and *Radix et Rhizoma Glycyrrhizae* (Licorice, Gancao). Single herbs in BZYQD, such as *Hedysarum multijugum* Maxim., *Angelicae Sinensis* Radix, and *Cimicifugae rhizoma*, have been reported to have protective effects against OP [13, 14]. However, the bioactive compounds and pharmacological mechanism of BZYQD in treating OPF remain unclear and warrant further investigation.

Traditional Chinese formulas contain numerous different herbs that may include multiple bioactive compounds and exhibit synergistic effects by acting on various targets [15]. Network pharmacology combines network biology and pharmacology and is a novel paradigm investigating the interactions among drugs, targets, and diseases regarding interconnected networks and biological pathways [16]. It has been used to discover the bioactive compounds in Yinqiao powder and Jiangzhi granule that interact with candidate proteins and biological pathways for COVID-19 [17] and non-alcoholic steatohepatitis [18], respectively.

**Fig. 1:**
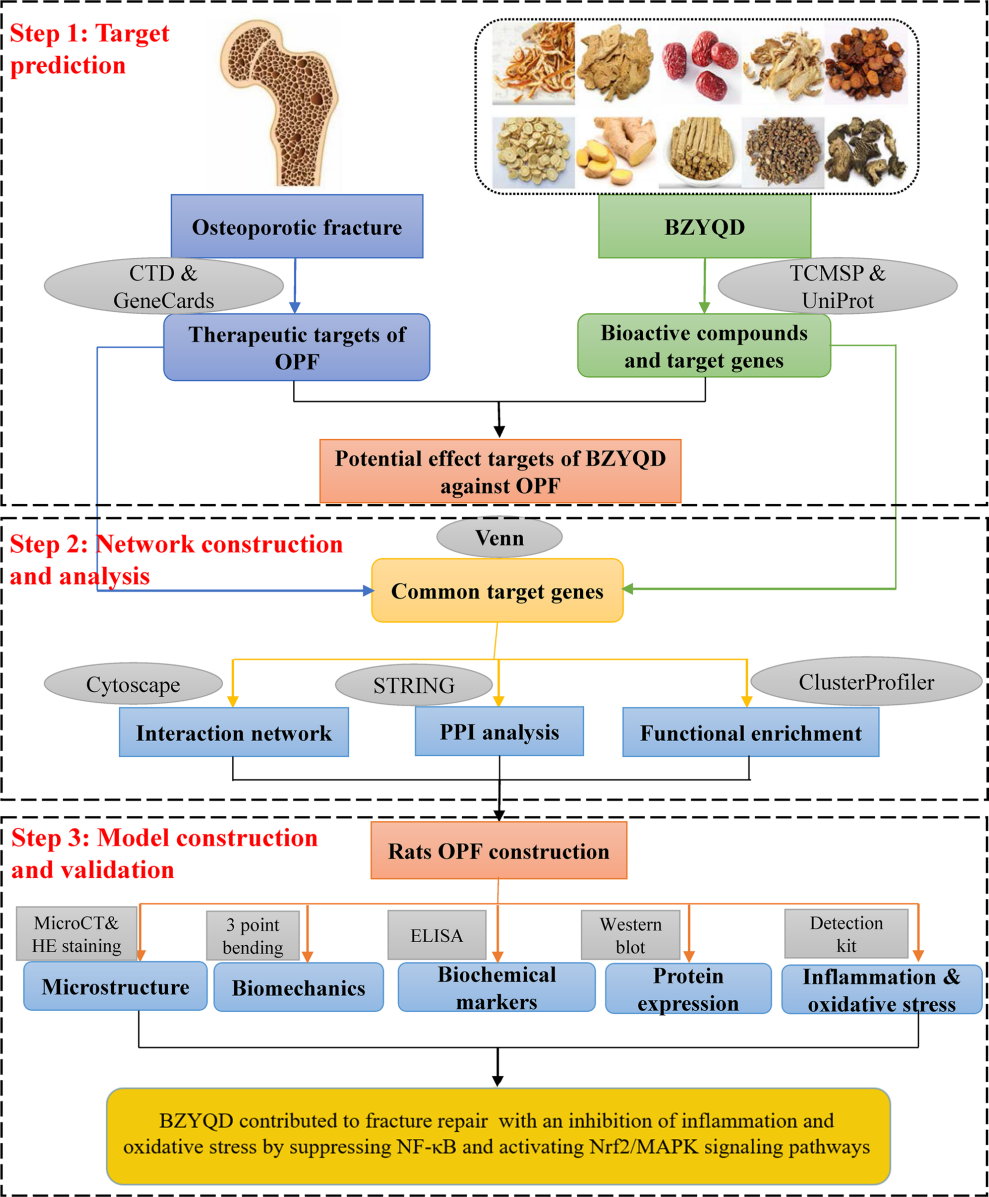
Workflow of the present study

The present study used network pharmacology to screen and discover bioactive compounds and potential therapeutic BZYQD targets for treating OPF. A BZYQD-bioactive compound-target gene-disease network was constructed, and functional enrichment analyses were performed. Furthermore, an OPF rat model treated with BZYQD was used to validate the findings of network pharmacology analyses. The workflow used in this study is illustrated in Fig. 1. Our results illuminate the therapeutic mechanisms of BZYQD involved in treating OPF and provide a basis for developing novel drugs against OPF.

## Materials and methods

### Screening bioactive compounds and targets of BZYQD

The single herbs, Atractylodes macrocephala Koidz. (Baizhu), Radix Bupleuri (Chaihu), Citrus reticulata (Chenpi), Jujubae Fructus (Dazao), *Angelicae Sinensis* Radix (Danggui), Codonopsis Radix (Dangshen), *Hedysarum multijugum* Maxim. (Huangqi), *Cimicifugae rhizoma* (Shengma), *Zingiber officinale* Roscoe (Shengjiang), and licorice (Gancao) were searched in the Traditional Chinese Medicine System Pharmacology and Analysis Platform databases (TCMSP, https://tcmspw.com/tcmsp.php) [19]. The oral bioavailability (OB) and drug-likeness (DL) values were set to > 30% and > 0.18, respectively, to screen candidate bioactive compounds [20]. Target genes of the bioactive compounds were obtained from TCMSP, and their official gene names were obtained from UniProt Knowledgebase (UniProtKB, https://www.uniprot.org/) [21].

### OPF target retrieval

The Comparative Toxicogenomics Database (CTD, http://ctdbase.org/) [22] and the Human Gene Database (GeneCards, https://www.genecards.org/) [23] were searched for potential therapeutic target genes for OPF using “Osteoporotic fracture” as the search term.

### Network construction and analysis

The potential BZYQD target genes in OPF treatment were identified by mapping the bioactive compound-related BZYQD target genes with those of the OPF-related using the VennDiagram package [24] in R 4.1.0 (https://www.r-project.org/). The interaction relationships between BZYQD-bioactive compounds and common target genes were determined, and an interaction network was constructed using Cytoscape software 3.8.2 (https://cytoscape.org/) [25].

### Construction of protein–protein interaction network

The Search Tool for the Retrieval of Interacting Genes (STRING V11, https://www.string-db.org/) [26] was used to construct a protein–protein interaction (PPI) network of common target genes. The “Homo sapiens” protein species were selected, and the “medium confidence (0.400)” was set as the lowest interaction threshold. The data generated from the PPI analysis were exported, and the PPI network was constructed using Cytoscape. The CytoNCA plugin [27] for Cytoscape was used to perform topological network analysis.

### Functional enrichment analysis of common target genes

Gene Ontology (GO) and the Kyoto Encyclopedia of Genes and Genomes (KEGG) signaling pathway enrichment analyses were performed to identify possible biological functions of the common target genes using the clusterProfiler package [28] in R software. The categories of biological processes, molecular functions, and cellular components were included in the GO terms. A Benjamini–Hochberg adjusted *p* value of < 0.05 indicated significantly enriched GO terms and KEGG pathways.

### Preparation of BZYQD

The BZYQD contained 20 g of *Atractylodes macrocephala Koidz*, 24 g of *Radix Bupleuri*, 12 g of *Citrus reticulata*, 20 g of *Ziziphus jujuba* Mill, 20 g of *Angelicae Sinensis Radix*, 30 g of *Codonopsis pilosula (Franch.) Nannf.*, 30 g of *Astragalus membranaceus* (Fisch.) Bunge, 12 g of *Cimicifugae rhizoma*, 42 g of *Zingiber officinale Roscoe*, and 30 g of *Radix et Rhizoma Glycyrrhizae.* Single herbs in the Chinese herbal BZYQD formula were obtained from the Wuxi Affiliated Hospital of Nanjing University of Chinese Medicine (Wuxi, China). In brief, the ten crushed herbs were mixed and soaked in 2L water at room temperature for 2 h and extracted by decocting twice for 30 min to obtain aqueous extracts. Then, the mixture was then filtered and concentrated to 1.04 g crude drug//mL as BZYQD for the experiments and stored at − 20 °C before use.

### Animals and BZYQD administration

Fifty female Sprague–Dawley (SD) rats (SPF grade, 8 months old; weight, 250–300 g) were purchased from Shanghai SLAC Laboratory Animal Co., Ltd. (Certificate No. SCXK (Hu) 2017-0005). They were adaptively fed for one week in standard squirrel cages (temperature, 22 ± 2 °C; humidity, 50–60%) with 12 h of circulating light. Regular pellet diet and water were available as needed. The Experimental Animal Ethics Committee of Wuxi Affiliated Hospital of Nanjing University of Chinese Medicine approved the methods used in this study (Approval No.: YKT2021032905-01).

The SD female rats were randomly divided into five groups (*n* = 10 per group): the control, model, and different dose of BZYQD intervention groups. All rats were anesthetized intraperitoneally with 2% sodium pentobarbital (80 mg/kg, Y0002194, Sigma-Aldrich, USA). Ovariectomy (OVX) was performed on the rats to induce OP [29]; bilateral incisions were made in the rat’s dorsal lower abdomen, followed by bilateral oophorectomy. In the control group, only a part of the soft tissue around the ovary was removed, and the incision was sutured. After 3 months of aging, all rats were anesthetized intraperitoneally with 2% sodium pentobarbital. We inserted the right femur using a Kirschner wire and modeled the fracture using a custom-made three-point bending device [30]. In the BZYQD-12.6, BZYQD-25.2, and BZYQD-50.4 groups, the rats were administered 12.6 g/kg/day, 25.2 g/kg/day, and 50.4 g/kg/day of BZYQD, respectively, via oral gavage for another 8 weeks, starting from the day after the OPF model establishment. The medium dose of BZYQD for rats in the BZYQD-25.2 group was calculated with a body surface area normalization method in the light of normal clinical dose using the following formula: Rats (g/kg) = [human dose (240 g crude drug/day)/human weight (60 kg)] × 6.3. Next, the low-dose and high-dose BZYQD were used as 1/2 and 2 times medium dose of BZYQD, respectively. Additionally, there were no side effects for the treatment found in this study. The rats in the model and control groups were administered the same volume of saline solution. At the endpoint, all rats were euthanized by administering an anesthetic overdose. All femurs were collected for follow-up evaluations. The peripheral blood of the rats was collected after anesthetization for serum biological index examination.

### Radiographic analysis

Radiographic images of the rat femur fracture sites were acquired using an X-ray system (PXLB7000, PERLOV, China) before sacrifice to assess the fracture healing status. The scoring criteria for fracture healing were as follows: 1 point indicated no healing, 2 points indicated no apparent callus formation, 3 points indicated partial fracture healing of the fracture, 4 points indicated that the fracture line and callus disappeared gradually, and 5 points indicated complete healing.

### Micro-computed tomography analysis

Harvested femurs were scanned using a micro-CT scanner with 35 μM resolution (MCT-III, ZKKS, China). Each group of pictures underwent three-dimensional reconstruction under the same conditions; morphometric analysis was performed using the evaluation software of the μCT system. The bone mineral density (BMD), bone surface density (BS/TV), bone volume fraction (BV/TV), trabecular number (Tb.N), trabecular separation (Tb.Sp), and trabecular density (Tb.Th) were measured.

### Biomechanical testing

Mechanical testing was performed using a three-point bending machine (INSTRON3382; Instron, USA). The femurs were placed at two fulcrums at a distance of 17 mm, and the load stress was applied at the midpoint of the callus area. The loading point was defined as the center of the fracture site. The measured biomechanical parameters included ultimate load, elastic modulus, and bending stress.

### Histological analysis

The hematoxylin and eosin (H&E) kit (BL700A) was purchased from Biosharp (China). Right femoral tissues were fixed in 10% formalin (E672001, Sangon, China) for one day, followed by decalcification in 9% formic acid. After, the specimens were dehydrated and embedded in paraffin. After sectioning (5-μm-thick), the slices were dewaxed using xylene and hydrated with gradient ethanol. Hematoxylin was used to stain the slices for 5 min. The slices were then differentiated using 1% hydrochloric acid. After counterstaining with eosin, the slices were dehydrated and permeated. An optical microscope was used to capture photographs of selected areas (ECLIPSE 80i, Nikon, Japan). The scoring criteria for fracture healing in rats were consistent with that in previous studies [31].

### ELISA assay

Peripheral blood was centrifuged (3000 rpm, 10 min), and the upper serum was harvested. Serum indicators related to bone metabolism, inflammation, and oxidative stress were measured using an ELISA assay. The following kits: alkaline phosphatase (ALP) (MM-0436R1), tartrate-resistant acid phosphatase (TRAP) (MM-0620R1), cross-linked telopeptide of type I collagen (CTXI) (MM-20171R1), osteocalcin (OC) (MM-20603R1), osteoprotegerin (OPG) (MM-0115R1), bone Gla protein (BGP) (MM-0700R1), irisin (MM-20578R1), IL-1β (MM-0047R1), IL-6 (MM-0190R1), tumor necrosis factor-alpha (TNF-α) (MM-0180R1), IL-10 (MM-0195R1), GSH (MM-0602R1), SOD (MM-0386R1), and malondialdehyde (MDA) (MM-0385R1), were obtained from MEIMIAN (China). We purchased calcium (RX301026R) and phosphorus kits (RX301537R) from RUIXIN (China). The biochemical bone markers, inflammation-related markers, and oxidative stress-related factors were measured according to the manufacturer’s instructions.

### Western blotting assay

For western blot analysis, proteins were extracted from femoral tissues using RIPA Buffer (20101ES60, Yeasen, China). We used the BCA kit (BI-WB005, SBJBIO, China) for protein quantification. After electrophoresis, the protein was loaded onto PVDF membranes (PW0034, Leagene, China). Next, 5% bovine serum albumin (BL-082, SBJBIO, China) was added to seal the membranes (37 °C, 60 min). The membranes were then immersed in primary antibodies (4 °C, overnight) and subsequently in anti-rabbit secondary antibodies (31,466, Invitrogen, USA) or anti-mouse secondary antibodies (S0002, Affinity, USA) at 37 °C for 60 min. Protein visualization was performed using an ECL reagent (GK10008, GlpBio, USA) on an eZwest Lite Auto Imaging System (Genscript, USA). The primary antibodies of Nrf2 (1:2000, AF0639), heme oxygenase 1 (HO-1) (1:2000, AF5393), phospho-IKB alpha (Ser32/Ser36, 1:2000, AF2002), IKB alpha (1:2000, AF5002), phospho-NF-kB p65 (Ser536, 1:2000, AF2006), NF-kB p65 (1:2000, AF5006), phospho-extracellular regulated protein kinases ½ (ERK1/2) (Tyr204, 1:2000, AF1014), ERK1/2 (1:2000, AF0155), phospho-p38 mitogen-activated protein kinase (MAPK) (Thr180/Tyr182, 1:500, AF4001), p38 MAPK (1:1000, AF6456), and GAPDH (1:20,000, AF7021) were obtained from Affinity (USA). GAPDH was used as the loading control.

### Statistical analysis

The measurement data are presented as the mean ± standard deviation. Statistical analysis was performed using the SPSS software (version 16.0). Each quantitative experiment was repeated at least thrice; multi-group comparisons were carried out using one-way ANOVA with Tukey’s test. For those with unequal variances, the Kruskal–Wallis H test was used. *p* < 0.05 indicated statistical significance.

## Results

### Bioactive compounds and target genes of BZYQD

Using the TCMSP database, 147 bioactive BZYQD compounds were identified, including 3 bioactive compounds in Atractylodes macrocephala Koidz., 11 in Radix Bupleuri, 5 in Citrus reticulata, 17 in Jujubae Fructus, 2 in Angelicae Sinensis Radix, 17 in Codonopsis Radix, 17 in Hedysarum Multijugum Maxim., 9 in Cimicifugae Rhizoma, 5 in Zingiber officinale Roscoe, and 89 in licorice. Besides, 130 potential target genes of these bioactive compounds were identified.

### Potential target genes of BZYQD in the treatment of OPF

**Fig. 2:**
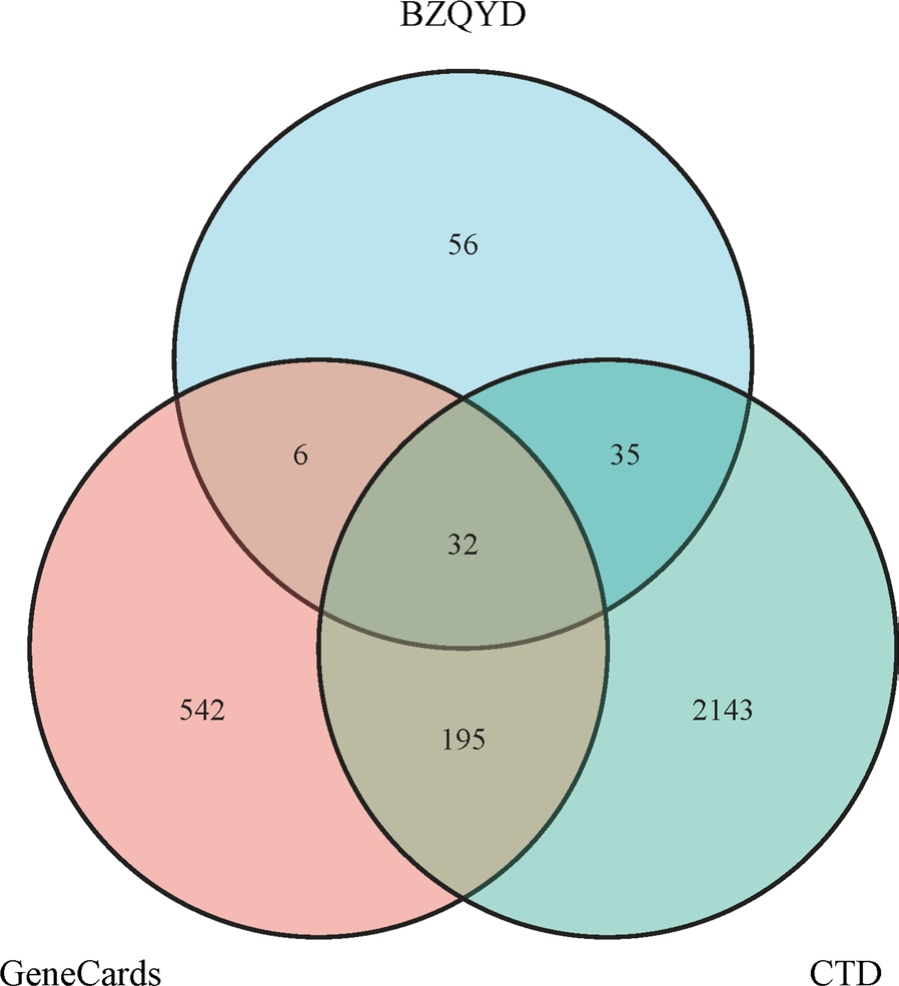
Venn diagram for BZYQD and OPF-related target genes

**Fig. 3:**
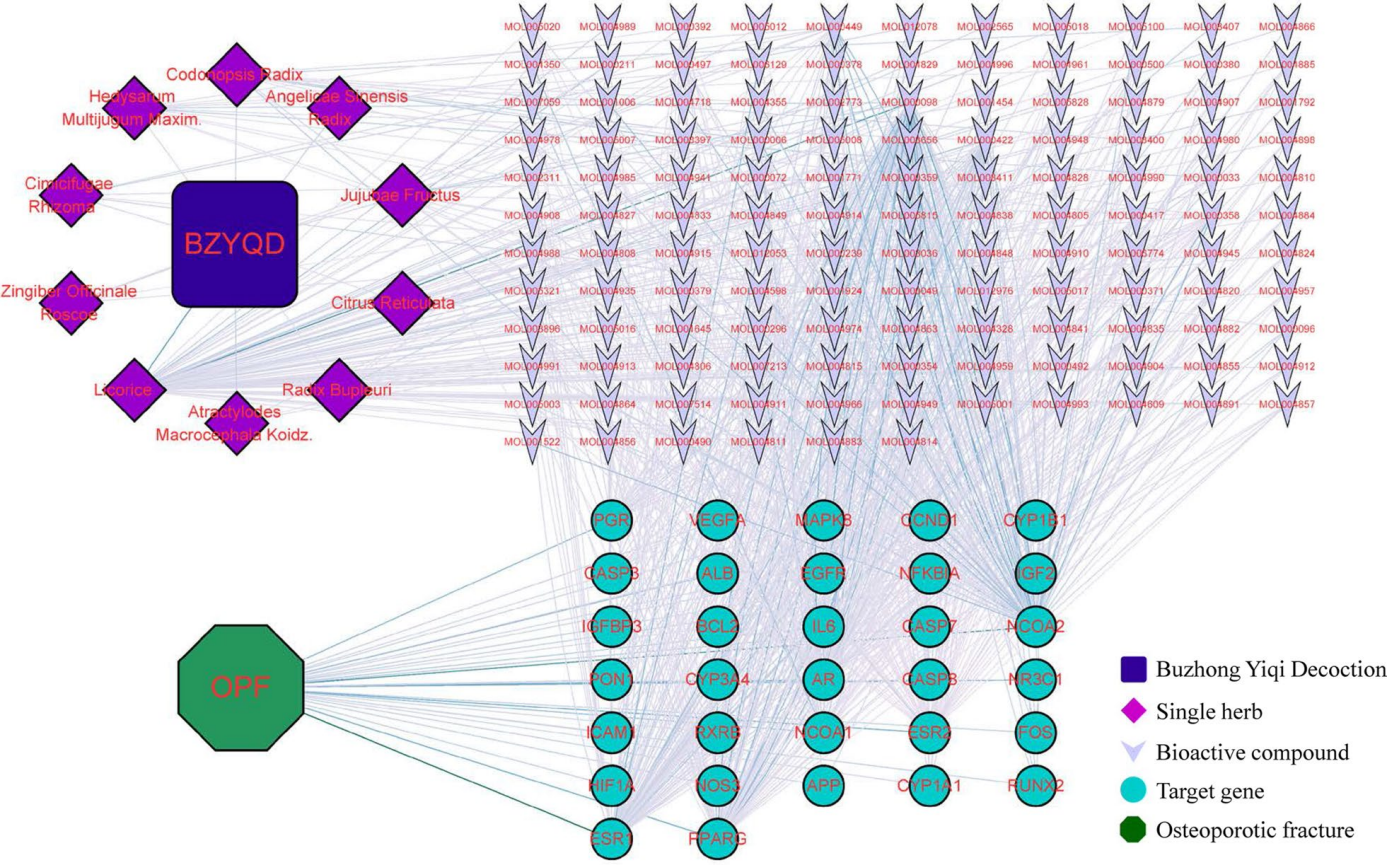
BZYQD-bioactive compound-target gene-OPF interaction network

A total of 775 and 2405 OPF-related target genes were identified by searching the GeneCards and the CTD databases, respectively. Finally, we found 32 common genes between the BZYQD-bioactive compound-target genes and OPF-related genes (Fig. 2). Subsequently, the “BZYQD-bioactive compound-target gene-OPF” network was constructed (Fig. 3). Of the 147 bioactive BZYQD compounds, 129 showed a direct therapeutic effect on OPF by acting on the 32 target genes. According to the gene count, the top 10 bioactive compounds in the network are listed in Table 1.

**Table 1:** The top 10 bioactive compounds in BZYQD

Bioactive compounds	MOL ID	Gene count	OB (%)	DL	Corresponding herbs
Quercetin	MOL000098	89	46.43	0.28	Radix Bupleuri, Jujubae Fructus, Licorice, *Hedysarum multijugum* Maxim
Kaempferol	MOL000422	36	41.88	0.24	Radix Bupleuri, Licorice, *Hedysarum multijugum* Maxim
Isorhamnetin	MOL000354	18	49.60	0.31	Radix Bupleuri, Licorice, *Hedysarum multijugum* Maxim
Stigmasterol	MOL000449	18	43.83	0.76	Radix Bupleuri, Jujubae Fructus, *Angelicae sinensis* Radix, Codonopsis Radix, *Cimicifugae rhizoma*, *Zingiber officinale* Roscoe
β-sitosterol	MOL000358	16	36.91	0.75	*Citrus reticulata*, Licorice, *Cimicifugae rhizoma*
7-Methoxy-2-methyl isoflavone	MOL003896	12	42.56	0.20	Codonopsis Radix, Licorice
Luteolin	MOL000006	12	36.16	0.25	Codonopsis Radix
Calycosin	MOL000417	10	47.75	0.24	Licorice, *Hedysarum multijugum* Maxim
Formononetin	MOL000392	10	69.67	0.21	*Hedysarum multijugum* Maxim
Nobiletin	MOL005828	8	61.67	0.52	*Citrus reticulata*

*OB* Oral bioavailability, *DL* Drug-likeness

### PPI network analysis

**Fig. 4:**
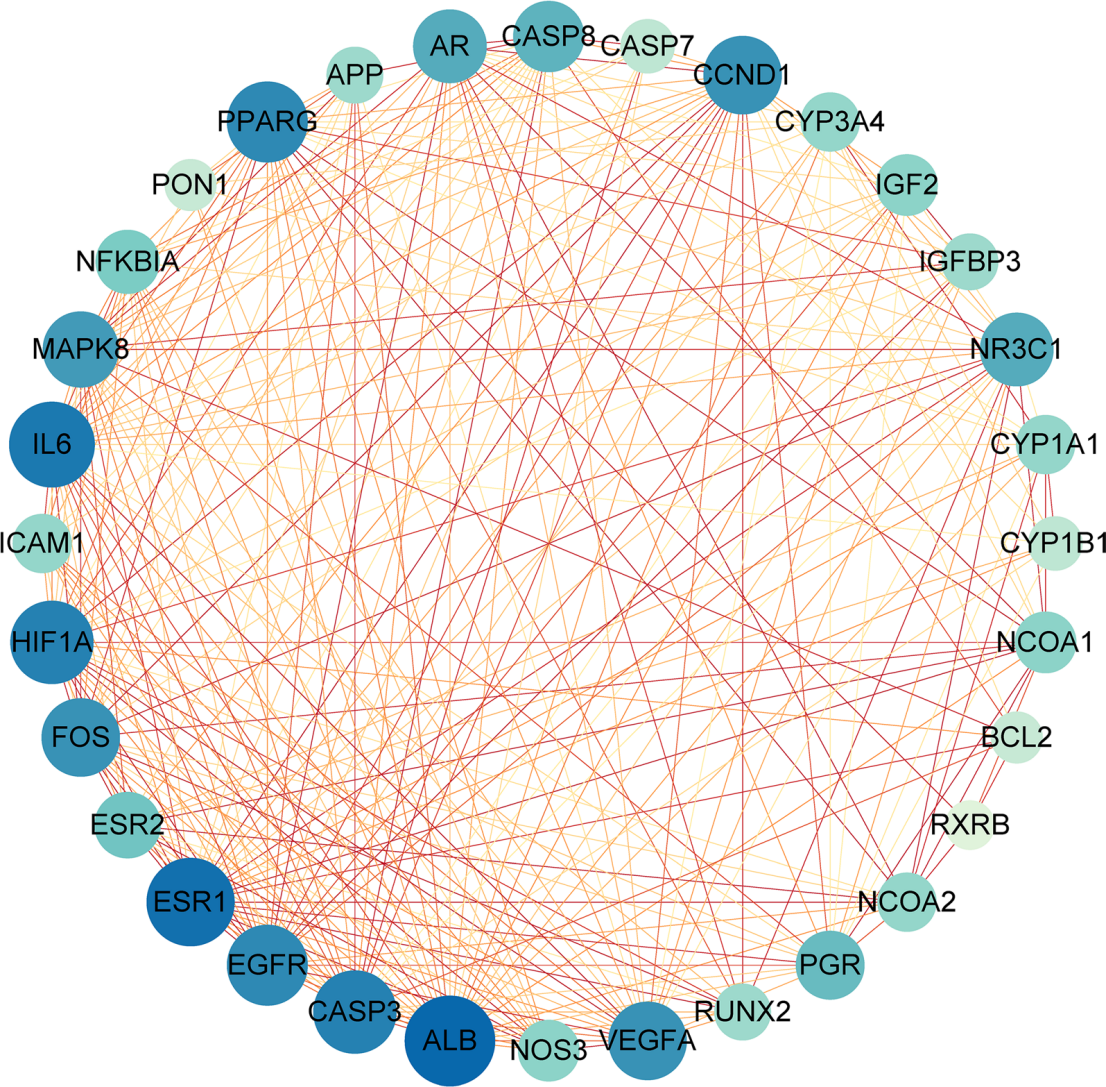
Protein–protein interaction network of common target genes

To comprehensively explore the pharmacological mechanism of BZYQD in treating OPF, we constructed a PPI network using the 32 common genes. The target gene PPI network consisted of 32 nodes and 272 edges. According to topological network analysis, PPI nodes with high degree values were considered important therapeutic targets for OPF treatment, including ALB, ESR1, IL6, MAPK8, and NFKB1A (Fig. 4).

### GO and KEGG enrichment analysis

**Fig. 5:**
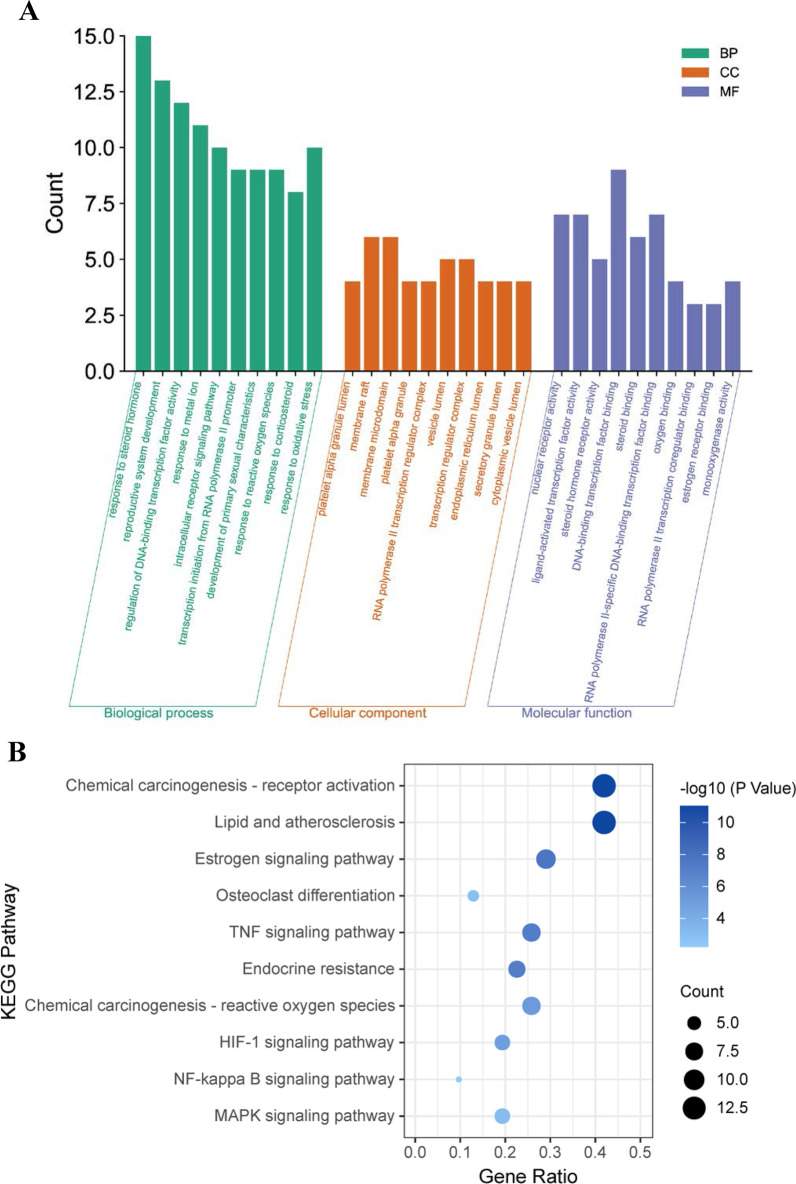
Functional enrichment analysis of common target genes. **A** The bar plot of enriched GO terms of common target genes. **B** The bubble plot of enriched KEGG pathways of common target genes

Functional enrichment analyses were performed to improve the biological understanding of the shared genes. Regarding biological processes, GO enrichment analysis showed that common genes were mainly involved in response to steroid hormones, regulation of DNA-binding transcription factor activity, intracellular receptor signaling pathway, response to reactive oxygen species, and response to oxidative stress. In the cellular component analysis, common genes were mainly associated with the platelet alpha granule lumen, RNA polymerase II transcription regulator complex, endoplasmic reticulum lumen, and secretory granule lumen. The significantly enriched molecular functions were steroid hormone receptor activity, oxygen binding, estrogen receptor binding, and monooxygenase activity (Fig. 5A). KEGG pathway enrichment analysis showed that treating OPF with BZYQD was mainly related to chemical carcinogenesis-receptor activation, lipid and atherosclerosis, estrogen signaling pathway, osteoclast differentiation, TNF signaling pathway, endocrine resistance, reactive oxygen species, HIF-1 signaling pathway, NF-κB signaling pathway, and MAPK signaling pathway (Fig. 5B).

### BZYQD promotes fracture repair in OPF rats

**Fig. 6:**
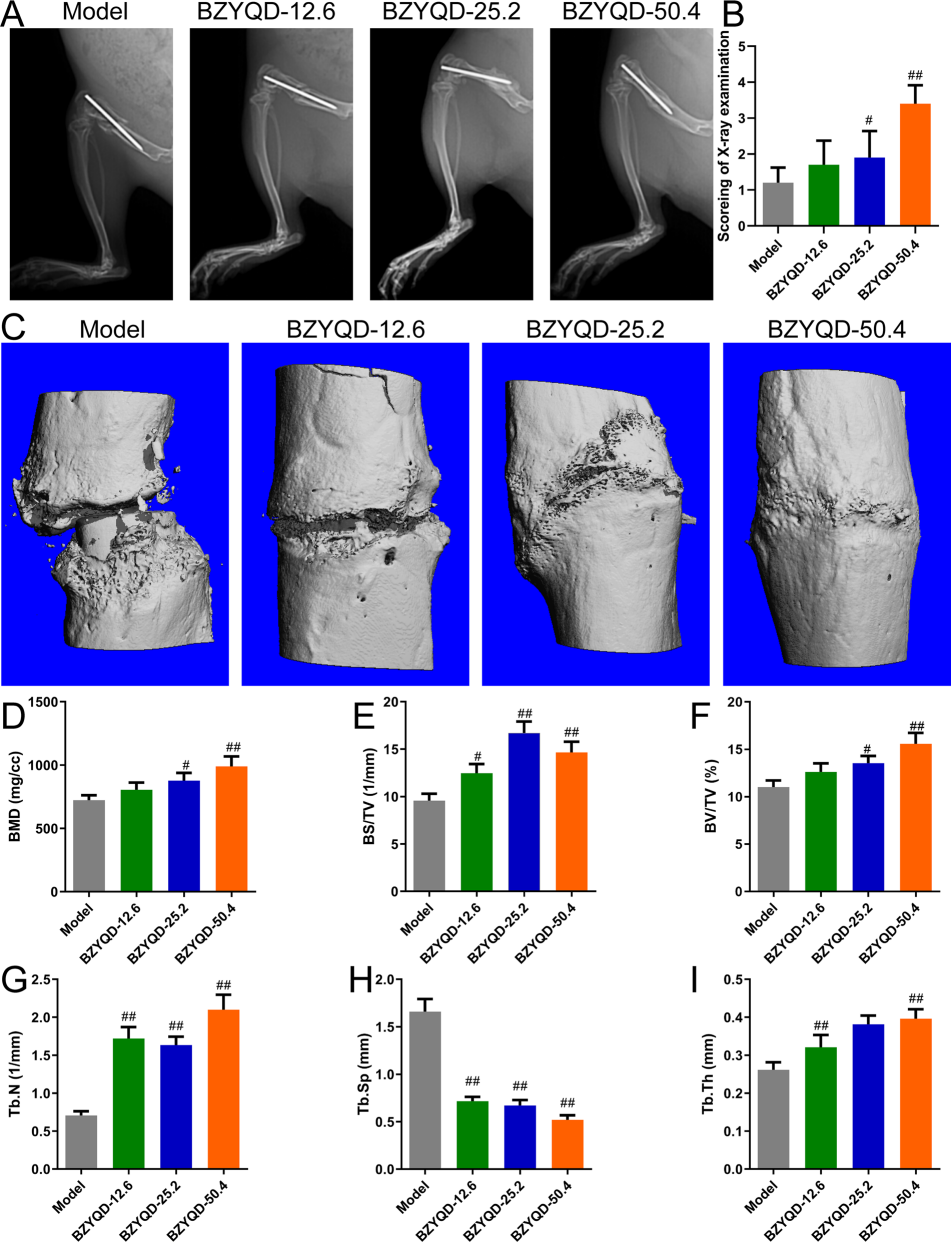
Treatment with BZYQD promotes fracture repair in OPF rats. **A**, **B** X-ray films and their X-ray examination scores (*n* = 10). **C** The photographs of micro-CT. **D**–**I** The bone mineral density (BMD) and microstructure micro-CT parameters (*n* = 3). ^#^*p* < 0.05, ^##^*p* < 0.01, versus model group

The fracture line in the model group was clear, and no or little callus formation was found; compared to the model group, the fracture line was still evident in the BZYQD-12.6 group, with small amounts of callus formation. BZYQD blurred the fracture line, facilitating callus formation dose-dependently (Fig. 6A). Moreover, BZYQD elevated the fracture healing scores dose-dependently (Fig. 6B, *p* < 0.05). Figure 6C shows micro-CT images. Micro-CT analysis revealed that each BZYQD dose strongly augmented BS/TV and Tb.N and repressed Tb.Sp in OPF rats, low and high BZYQD doses significantly elevated Tb.Th in OPF rats; medium and high BZYQD doses significantly advanced BMD and BV/TV in OPF rats (Fig. 6D–I, *p* < 0.05).

### BZYQD ameliorated the biomechanical property and femoral microarchitecture in OPF rats

**Fig. 7:**
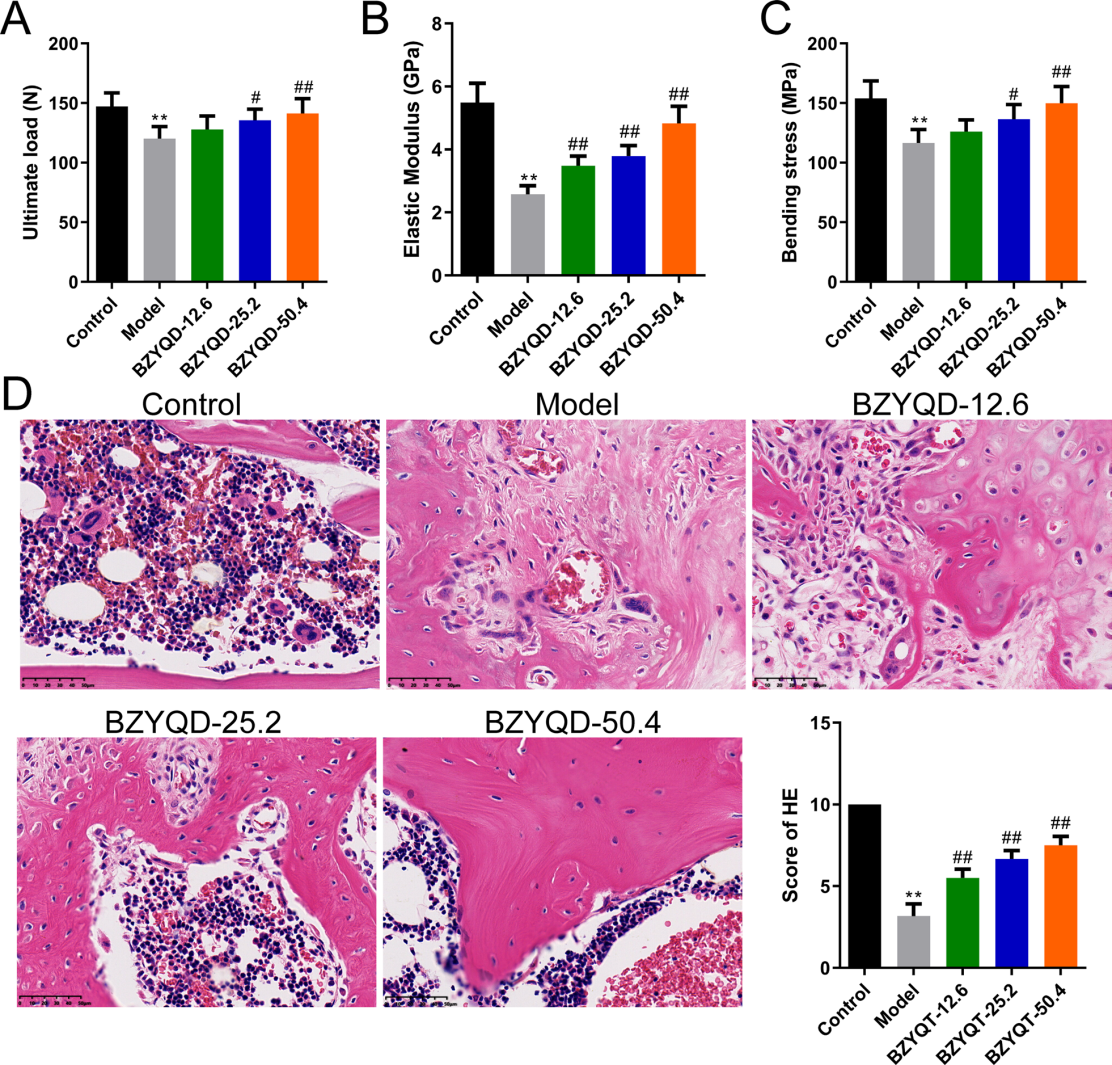
BZYQD ameliorated the biomechanical property and femoral microarchitecture in OPF rats. **A**–**C** The results of the three-point bending examination of ultimate load, elastic modulus, and bending strength in different groups (*n* = 6). **D** The representative images and scores of H&E staining (× 400) (*n* = 6). **p < 0.01, versus control group; ^#^*p* < 0.05, ^##^*p* < 0.01, versus model group

Figure 7A–C shows that the ultimate load, elastic modulus, and bending stress in the model group were lower than those in the control group; BZYQD dose-dependently upregulated the ultimate load, elastic modulus, and bending stress in OPF rats (*p* < 0.05). H&E staining results revealed that the femoral tissue of the rats in the control group was mature bone tissue, and the trabecular bone was evenly distributed and closely arranged; the femoral tissue of the rats in the model group was mainly cartilage, with more fibrous tissue, and less bone trabecular and medullary cavity. In the BZYQD-12.6 group, the femoral tissue was mainly cartilage, with increased trabecular bone and osteoblast number. The femoral tissue of the rats in the BZYQD-25.2 group was dominated by cartilage, with a small amount of immature bone; the trabecular bone was evenly distributed, and the number was more than that in the BZYQD-12.6 group. The immature bone was dominant in the femoral tissue of the rats in the BZYQD-50.4 group, and the trabecular bone was evenly distributed and abundant, with an apparent medullary cavity (Fig. 7D). BZYQD augmented the scoring of H&E staining of the femoral tissue in OPF rats in a dose-dependent manner (Fig. 7D, *p* < 0.01).

### Effect of BZYQD on the serum biochemical indexes in OPF rats

**Fig. 8:**
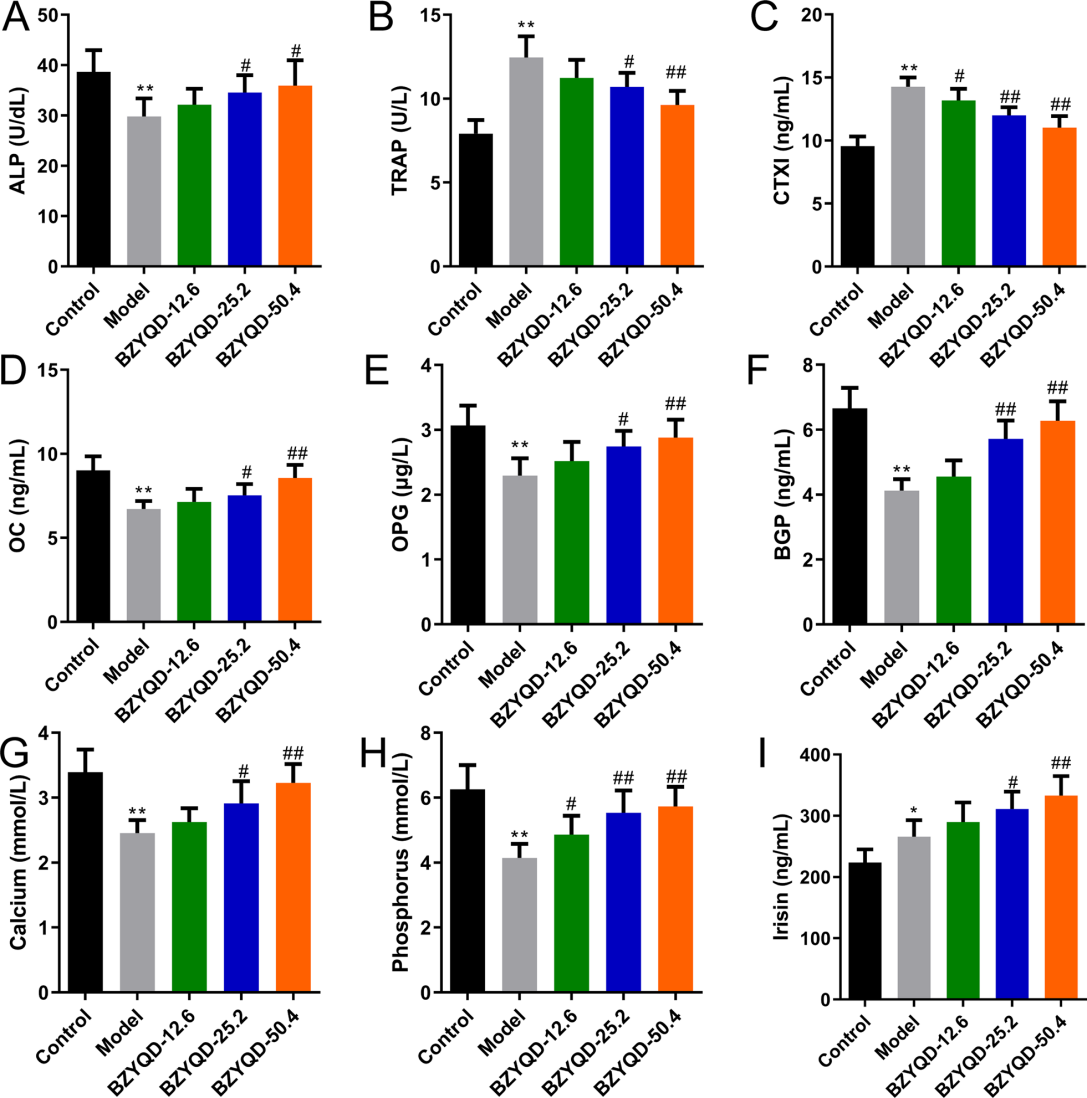
BZYQD increases the levels of bone formation indicators and reduces bone absorption indicators in OPF Rat. **A** ALP, **B** TRAP, **C** CTXI, **D** OC, **E** OPG, **F** BGP, **G** Calcium, **H** Phosphorus, and **I** Irisin (*n* = 6). *p < 0.05, **p < 0.01, versus control group; ^#^*p* < 0.05, ^##^*p* < 0.01, versus model group

Compared to the control group, the levels of ALP, OC, OPG, BGP, calcium, and phosphorus in the serum of the model group were downregulated. In contrast, TRAP, CTXI, and irisin levels were upregulated (Fig. 8A–I, *p* < 0.05). BZYQD unexpectedly enhanced the levels of irisin, ALP, OC, OPG, BGP, calcium, and phosphorus, while suppressing those of TRAP and CTXI in OPF rats (Fig. 8A–I, *p* < 0.05).

### BZYQD relieved inflammation and oxidative stress level in serum and femoral tissue of OPF rats

**Fig. 9:**
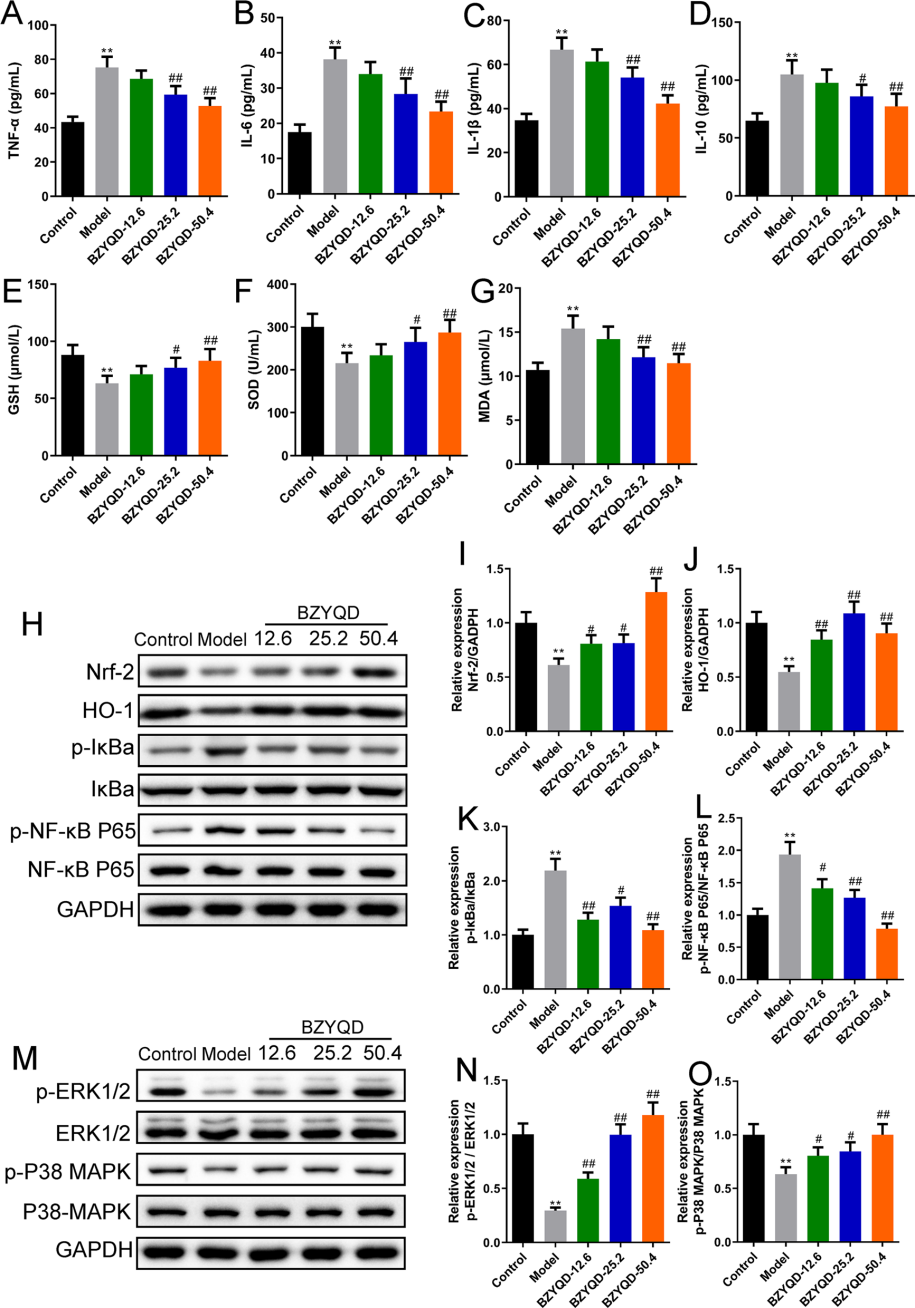
BZYQD suppressed the activation of NF-κB and activated Nrf2/MAPK signaling pathways. **A** TNF-α, **B** IL-6, **C** IL-1β, and **D** IL-10 (*n* = 6). BZYQD upregulated the **E** GSH and **F** SOD and downregulated the **G** MDA (*n* = 6). **H** Representative Western blot bands and the results of semi-quantitative analysis of **I** Nrf-2, **J** HO-1, **K** p-IKBα/IKBα, and **L** p-NF-κB/NF-κB (*n* = 3). **M** The Western blot bands and the results of semi-quantitative analysis of **N** p-ERK1/2/ERK1/2 and **O** p-P38 MAPK/P38 MAPK (*n* = 3). **p < 0.01, versus control group; ^#^*p* < 0.05, ^##^*p* < 0.01, versus model group

As mentioned above, the identified candidate target genes of BZYQD were mainly enriched in the ROS, TNF signaling pathway, NF-κB signaling pathway, and MAPK signaling pathway. Therefore, we focused on exploring inflammation and oxidative stress levels in the related pathway's serum and protein expression levels. We detected inflammation- and oxidative stress-related markers in the serum of OPF rats using ELISA. Nrf2, HO-1, NF-κB, ERK, and MAPK protein expressions were analyzed using western blotting. BZYQD dose-dependently reduced TNF-α, IL-6, IL-1β, IL-10, and MDA levels and augmented those of GSH and SOD in the serum of OPF rats (Fig. 9A–G, *p* < 0.05). At the molecular level, we also demonstrated that BZYQD in each dose group strengthened the Nrf2 and HO-1 levels, as well as the ratios of p-ERK1/2/ERK1/2 and p-P38 MAPK/P38 MAPK. In contrast, it significantly repressed the ratios of p-IKBα/IKBα and p-NF-κB P65/NF-κB P65 in the femoral tissue of OPF rats (Fig. 9H–R, *p* < 0.05).

## Discussion

OPFs are associated with a high risk of disability and mortality, particularly in postmenopausal women. Approximately 10% of these patients experience fracture healing delay or even failure in fracture healing, which significantly affects the patient’s quality of life and adds to their economic burden [32]. Moreover, the presently available drugs for treating osteoporotic fractures may cause side effects, which are compromised by long-term application [33]. Therefore, the discovery of novel therapeutic targets and the development of novel drugs for efficient and safe therapy of osteoporotic fractures are urgently warranted [34]. BZYQD is a widely used Chinese herbal formula for treating complicated diseases, including female stress urinary incontinence [35], sexual dysfunction [36], and OPF. However, the ingredients and therapeutic mechanisms of OPF require clarification. In the present study, we integrated network pharmacology and OPF animal model validation to explore the main bioactive BZYQD compounds and their potential targets against OPF.

The main bioactive compounds and target genes of BZYQD in OPF were identified using network pharmacology analysis. We observed that BZYQD might exert its therapeutic effects in OPF through its main bioactive compounds, including quercetin, kaempferol, isorhamnetin, stigmasterol, β-sitosterol, 7-Methoxy-2-methyl isoflavone, luteolin, calycosin, formononetin, and nobiletin. Moreover, most of these compounds have been reported to be associated with OP and OPF. Sun et al. observed that quercetin could reduce OP by regulating glucose metabolism and inhibiting lipid metabolism [37]. Kaempferol has been reported to promote osteogenesis in bone marrow mesenchymal stem cells [38]. Marahatha et al. reported that stigmasterol and β-sitosterol have similar pharmacokinetic properties in inflammatory diseases, including OP and metabolic abnormalities [39]. By regulating the ERK/Lrp-5/GSK-3β signaling pathway, luteolin shows antioxidant and anti-inflammatory effects against glucocorticoid-induced OP [40]. Singh et al. reported that formononetin led to predominant runt-related transcription factor 2 and osteocalcin localization at the bone injury site and promoted fracture repair [41]. These findings suggest that the bioactive BZYQD compounds might exhibit antioxidant and anti-inflammatory effects on OPF and promote bone formation. The present study’s results showed that BZYQD decreased pro-inflammatory cytokines and oxidative stress in OPF rats, partly consistent with the results of previous studies.

GO enrichment analysis revealed that the common genes were involved in response to steroid hormones, reactive oxygen species and oxidative stress, transcription regulation, and estrogen receptor binding, showing that the therapeutic effects of BZYQD may be associated with transcriptional target gene regulation, resistance to oxidative stress, and various hormone-related pathways. KEGG pathway analysis revealed that BZYQD might produce therapeutic effects via multiple signaling pathways, including the TNF, NF-κB, and MAPK signaling pathways. TNF-α is a pleiotropic cytokine with pivotal effects in mediating immune response, inflammation, cell survival and proliferation, metabolic processes in healthy organisms, and disease pathogenesis [42]. The TNF signaling pathway plays an essential role in the osteogenic differentiation of mesenchymal stem cells by affecting the inflammatory microenvironment, which may be a potential therapeutic target for complex bone fracture diseases [43]. It has been noted that NF-κB and MAPK signaling pathways play essential roles in bone formation and resorption. In addition, agents that target the NF-κB and MAPK signaling pathways may exert beneficial effects on OPF [44].

To explore the potential molecular mechanism of BZYQD on OPF, we established a rat OPF model and evaluated the fracture repair indices. BMD can reflect the state of bone turnover and can be used to predict the risk of fracture and diagnosis of OP [45]. In the present study, we measured fracture site healing conditions using micro-CT by X-ray and bone microstructure. The current results indicate that BS/TV, BV/TV, Tb.N, Tb.Sp, and Tb.Th may be sensitive variables for the early detection of bone trabecular structural changes, which can forecast OP development [46]. This study pointed out that BZYQD blurred the fracture line, facilitated callus formation, enhanced BMD, and eased the bone microstructure in OPF rats.

Furthermore, BZYQD dose-dependently enhanced fracture healing, supported by biomechanical testing and H&E staining results. We then detected OP-related indicators in the serum of the OPF rats. BZYQD unexpectedly increased irisin, ALP, OC, OPG, BGP, calcium, and phosphorus levels, while suppressing those of TRAP and CTXI in OPF rats, suggesting that BZYQD is beneficial for maintaining the metabolic balance of osteoblasts and osteoclasts. The initiation and development of OPF symptoms are usually accompanied by oxidative stress damage, which impacts fracture healing [47]. Changes in GSH, MDA, and SOD expression levels are closely related to the severity of oxidative stress injury [48]. Nrf2/HO-1 has a significant positive role in oxidative damage and body protection and is considered one of the essential mechanisms of intracellular antioxidant stress [49]. Some studies have found that the Nrf2/HO-1 pathway plays a considerable role in the pathogenesis of postmenopausal OP. Its activation can enhance the inhibitory effect of endogenous antioxidants on ROS, promoting bone growth regeneration [50]. Moreover, oxidative stress accelerates cellular senescence and death by activating many signaling pathways in vivo, including NF-κB and MAPK pathways [51, 52]. A clinical study has confirmed the increase in inflammatory factors in postmenopausal patients with OP, particularly IL-1β, IL-6, and TNF-α [53]. By conducting ELISA and western blotting, we found that BZYQD dose-dependently reduced the serum TNF-α, IL-6, IL-1β, and IL-10 levels, as well as the p-IKBα/IKBα and p-NF-κB P65/NF-κB P65 ratio in the femoral tissue. In contrast, it augmented the serum GSH and SOD levels and the protein levels of Nrf2 and HO-1 in OPF rats. Therefore, the ameliorating effect of BZYQD on OPF rats is associated with the Nrf2/HO-1 and NF-κB pathways. In addition, the MAPK signaling pathway is involved in bone remodeling through osteoblast differentiation [54]. Researchers elucidated that calcitonin restrained apoptosis and inflammation in LPS-mediated chondrocytes by modulating the MAPK/NF-κB pathway [55]. Another study suggested that *Pleurotus sajor-caju* (Fr.) Sing β-1,3-Glucanoligosaccharide (*Ps*-GOS) promotes bone formation by inducing osteoblast proliferation, differentiation, and mineralization via the MAPK pathway [56]. Our study reported that BZYQD in each dose group strengthened the p-ERK1/2/ERK1/2 and p-P38 MAPK/P38 MAPK ratios in the femoral tissue of OPF rats. In the present study, we observed that BZYQD significantly restored the activation of -NF-κB signaling and regulated that of Nrf2/MAPK signaling in OPF rats. This finding shows that BZYQD exerts therapeutic effects on inflammation and oxidative stress in OPF through its main bioactive compounds (such as quercetin, kaempferol, and luteolin) by modulating the NF-κB and Nrf2/MAPK signaling pathways.

Although some essential preliminary findings were obtained in this study, further bioinformatics and animal/cell studies with gain/loss-of-function experiments are needed, as well as the liquid chromatography mass spectrometry or High-performance liquid chromatography analysis should be performed to validate the preparation of BZYQD. The advantage of the multi-component and multi-target regulation of TCM compounds is the hope of promoting fracture repair in OPF. With the gradual refinement of the OPF model, long-term intervention studies of BZYQD should be carried out in a model that can reflect the entire process of bone remodeling, inflammation, and oxidative stress, which will help to systematically reveal the effective components of BZYQD and its mechanisms of regulating inflammation and oxidative stress and promoting fracture repair.

## Conclusion

In the present study, we found that BZYQD might exert its therapeutic effects on inflammation and oxidative stress in OPF by suppressing NF-κB signaling and activating Nrf2/MAPK signaling pathways. Our results may help elucidate the therapeutic mechanism of BZYQD against OPF and provide potential bioactive compounds and therapeutic targets for its treatment.

## Data Availability

The data used and/or analyzed during the current study are available from the corresponding author upon reasonable request.
